# Plasticity of primary microglia on micropatterned geometries and spontaneous long-distance migration in microfluidic channels

**DOI:** 10.1186/1471-2202-14-121

**Published:** 2013-10-13

**Authors:** Susanna Amadio, Adele De Ninno, Cinzia Montilli, Luca Businaro, Annamaria Gerardino, Cinzia Volonté

**Affiliations:** 1 Santa Lucia Foundation/CNR-Cellular Biology and Neurobiology Institute, Via del Fosso di Fiorano 65, 00143 Rome, Italy; 2 Department of Anatomy, Histology, Forensic Medicine and Orthopedics, University La Sapienza, Rome, Italy; 3 CNR-Institute for Photonics and Nanotechnology, Via Cineto Romano 42, 00156 Rome, Italy

**Keywords:** Confocal analysis, Long distance migration, Microglia plasticity, Microfabrication, Time-lapse microscopy

## Abstract

**Background:**

Microglia possess an elevated grade of plasticity, undergoing several structural changes based on their location and state of activation. The first step towards the comprehension of microglia’s biology and functional responses to an extremely mutable extracellular milieu, consists in discriminating the morphological features acquired by cells maintained *in vitro* under diverse environmental conditions. Previous work described neither primary microglia grown on artificially patterned environments which impose physical cues and constraints, nor long distance migration of microglia *in vitro*. To this aim, the present work exploits artificial bio-mimetic microstructured substrates with pillar-shaped or line-grating geometries fabricated on poly(dimethylsiloxane) by soft lithography, in addition to microfluidic devices, and highlights some morphological/functional characteristics of microglia which were underestimated or unknown so far.

**Results:**

We report that primary microglia selectively adapt to diverse microstructured substrates modifying accordingly their morphological features and behavior. On micropatterned pillar-shaped geometries, microglia appear multipolar, extend several protrusions in all directions and form distinct pseudopodia. On both micropatterned line-grating geometries and microfluidic channels, microglia extend the cytoplasm from a roundish to a stretched, flattened morphology and assume a filopodia-bearing bipolar structure. Finally, we show that in the absence of any applied chemical gradient, primary microglia spontaneously moves through microfluidic channels for a distance of up to 500 μm in approximately 12 hours, with an average speed of 0.66 μm/min.

**Conclusions:**

We demonstrate an elevated grade of microglia plasticity in response to a mutable extracellular environment, thus making these cells an appealing population to be further exploited for lab on chip technologies. The development of microglia-based microstructured substrates opens the road to novel hybrid platforms for testing drugs for neuroinflammatory diseases.

## Background

Microglia are part of the immune system being the resident macrophages of the brain and spinal cord, and were first discovered and defined by Pio Del Rio Hortega [1] as an independent cellular phenotype abundantly present in the central nervous system (CNS). Microglia are of mesodermal origin and possess an elevated grade of plasticity, undergoing a variety of structural changes based on their location and particular state of activation. Unlike other cells in the brain, they are extremely versatile and dynamic [2]. They have the capacity to sense and adjust to the microenvironment, to migrate, proliferate and phagocytose. During early development, microglia enter the brain using vessels and white matter tracts as guiding structure for migration, and then disperse throughout the CNS occupying a defined territory [3]. Late in development, they transform from an amoeboid morphology into a branched, ramified phenotype composed of long, constantly extending, shrinking and re-growing processes with small, fairly motionless cell bodies, known today as surveilling microglia. These shape-shifting cells constitute about 20% of the total glial cell population within the adult brain and act as the first and main form of active immune defense in the CNS, by frequently scavenging for pathogens, damaged or dead cells and misfolded proteins, but also trimming away weak or damaged synapses between neurons [4-6]. Indeed, after a pathological event, microglia respond to the environment by undergoing profound transition in morphological appearance, motility and state of activation, and reacquiring an amoeboid shape similar to the one observed early in development. This high level of plasticity is required to fulfill the vast variety of immunological functions that microglia perform, as well as for maintaining homeostasis within the brain [2,7-12].

The first step towards the comprehension of microglia’s biology consists in discriminating if different morphological features can be acquired *in vitro* when microglia are cultured on diverse surface topography that are mimetic of an *in vivo* resembling three-dimensional (3D) environment. To this aim, we employed microstructured pillar-shaped and line**-**grating geometries produced on poly(dimethylsiloxane) (PDMS) by standard soft lithography techniques, and two-layer microfabrication photolithography. While the use of artificial bio-mimetic microtextured substrates and microfluidic devices [13] was mostly exploited for cancer cells [14-16] and neurons [17,18], only few studies were performed so far on microglia [19,20].

## Results

### Microglia spontaneously adjust to micropatterned structures

**Figure 1:**
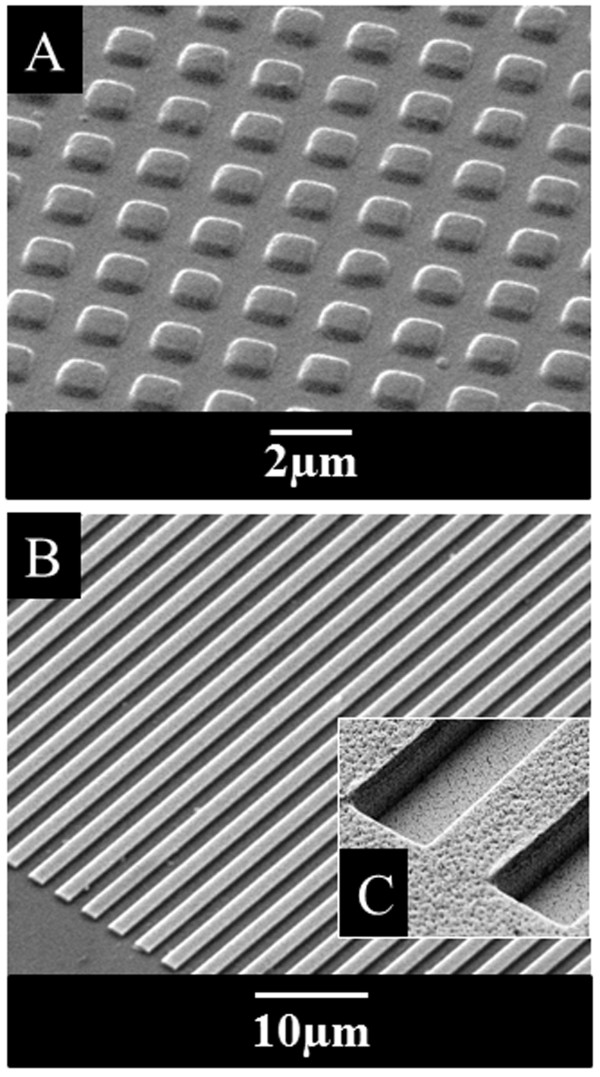
**Micropatterned structures serving for primary microglia culturing.** Microtopographic silicon masters are realized to replica-mold substrates on PDMS with pillar-shaped and line-grating geometries (width: 1500 nm, pitch: 3 μm, height: 550 nm). **A**. Scanning electron microscope (Zeiss EVO) images of PDMS pillar- shaped substrates. **B**. Scanning electron microscope images of Si master with line-grating structures. **C**. PDMS line-gratings at high magnification.

**Figure 2:**
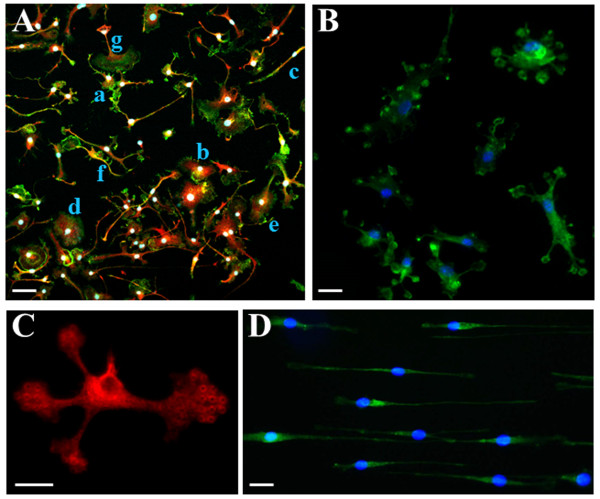
**Microglia adjust to micropatterned structures. A**. Primary mouse microglia are cultured on plastic dishes and subjected to immunofluorescence and confocal analysis with phalloidin (green) plus P2Y12 receptors antiserum (red) and Hoechst (blue), scale bar = 50 μm. **B**-**C**. Primary microglia are cultured on pillar microstructures subjected to fluorescence and confocal analysis with phalloidin (green) plus Hoechst (blue), scale bar = 20 μm **(B)**, or immunofluorescence with paxillin (red), scale bar = 10 μm **(C)**. **D**. Primary mouse microglia are cultured on line-grating geometries and subjected to fluorescence and confocal analysis with phalloidin (green) plus Hoechst (blue), scale bar = 20 μm.

The first aim of our work is to identify some experimental conditions that could distinguish the morphological states of microglia *in vitro*, since the variety of changes that microglia undergo *in vivo* depends on the heterogeneity of the environment [21-23]. Due to the extreme variability of microglial immortalized cell lines (such as N9 or BV-2 cells), we mostly used primary microglia dissociated cultures from rat and mouse cerebral cortex [24] plated on micropatterned pillar-shaped (Figure 1A) and line grating (Figure 1B,C) substrates. Figure 2A illustrates primary microglia cultured on plastic dishes coated with fibronectin, a condition commonly adopted *in vitro* for motility studies. Double fluorescence confocal analysis performed with phalloidin (a marker for filamentous actin, in green) and P2Y12 receptor antiserum (a marker for microglia, in red) [25] shows the heterogeneous morphological features of microglia in culture, likely representing different functional states (surveillance, scavenge, migration, phagocytosis, antigen presentation, cytotoxicity). Indeed, we simultaneously observe roundish cell bodies with single, long processes enriched by short and tiny branches (a); asymmetrical “hairy” cells with miniature processes (b); elongated “rod-like” cell bodies with no or few branches (c); amoeboid cells (d); cells crowned by lamellipodia (e), or possessing several filopodia (f) or fluffy fan-like cytoplasmic protrusions (g). When instead cultured on biocompatible microstructured PDMS substrates, microglia undergo a remarkable homogeneous metamorphosis. On pillar-shaped geometries (Figures 1A and 2B, 2C), the majority of cells appears multipolar, with protuberances spreading out from roundish or fairly oblongated cell bodies, forming button-shaped lamellipodia or pseudopodia. These protrusions furthermore highlight the subjacent pillar structures (phalloidin staining, panel B or paxillin, panel C), and are likely to reflect points of membrane adhesion to the underlying substrate. On line-grating geometries (Figures 1B,C and 2D), microglia become longitudinally flattened on the substrate and show a filopodia-bearing elongated bipolar structure [26].

### Morphometric analysis of microglia on micropatterned structures

**Figure 3:**
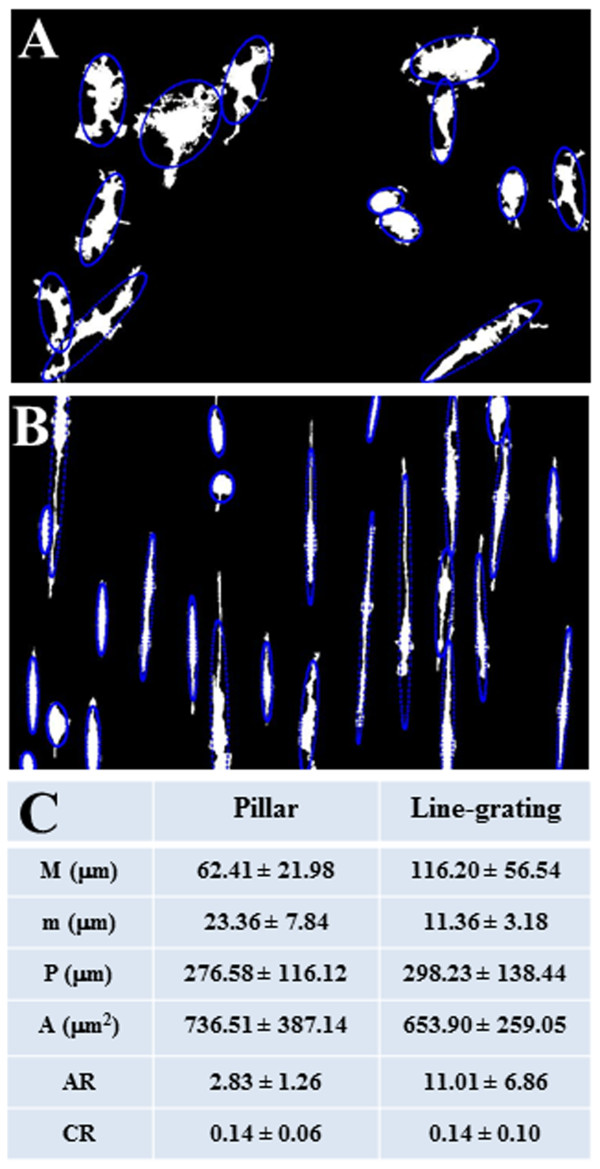
**Morphometric analysis of microglia on micropatterned structures.** Morphometric analysis of primary microglia cultured on pillar microstructures **(A)** and line-grating geometries **(B)** is performed using a customized Matlab code **(C)**. Values are expressed as mean ± SD, with n = 14 cells on pillars or line-grating structures. A significative difference is obtained for all parameters by Mann–Whitney test (p < 0.0003). “M” indicates major axis and “m” minor axis of cell’s fitted ellipse (panels **A**, **B**), “A” cell area, “P” cell perimeter, “AR” aspect ratio, “CR” circularity ratio.

These morphologic observations are confirmed by quantitative analysis performed importing the fluorescence and confocal microscopy images into Matlab. We adopted circularity ratio (CR) and axis ratio (AR) shape descriptors. CR defines the ratio between the area of the shape of interest and the area of a circle having the same perimeter [27]. This parameter represents how the shape of interest differs from a circle, and it is expressed as CR = 4πAP^-2^, where “A” is the cell area and “P” the cell perimeter length. AR defines the ratio between the major axis (M) and the minor axis (m) of the cell’s fitted ellipse [28], and represents a measure of how the shape of interest is “elongated”. In detail, we observe that on pillar (Figure 3A,C) or line-grating (Figure 3B,C) geometries, the average M, m and AR of microglia are different, remaining approximately constant the average P, A and CR values. Respectively on pillar or line-grating geometries, we find that M is 62.41 ± 21.98 μm or 116.20 ± 56.54 μm, m is 23.36 ± 7.84 μm or 11.36 ± 3.18 μm, AR is 2.83 ± 1.26 or 11.01 ± 6.86 (Figure 3C).

### Morphological transition of microglia in microfluidic devices

**Figure 4:**
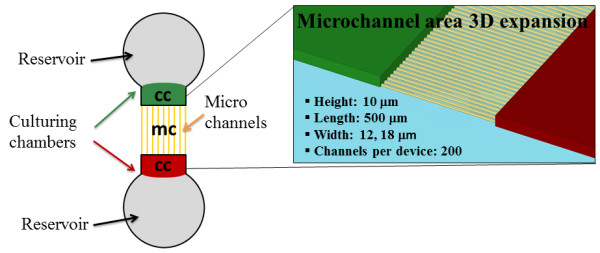
**Microfluidic structure adopted for primary microglia culturing.** Schematic representation of the microfluidic device used for the microglia motility experiments. Reservoirs, culturing chambers (cc) and microchannels (mc) areas are highlighted.

**Figure 5:**
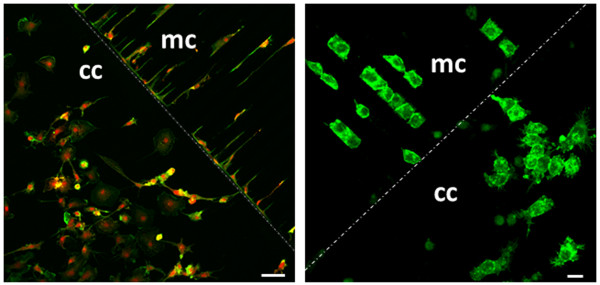
**Morphological transition of microglia in microfluidic devices.** Primary rat microglia (left panel) and N9 microglia cell line (right panel) are maintained in culture on microfluidic devices for 24 hours and subjected to immunofluorescence and confocal analysis with phalloidin (green) plus P2Y12 receptors antiserum (red) (left panel, scale bar = 50 μm) or fluorescence with phalloidin (right panel, scale bar = 20 μm). mc indicates the microchannels areas and cc shows the culturing chambers, as schematically depicted in Figure 4.

We then asked if the elongated bipolar structure of microglia on line-grating geometries might convert into propensity for instance to motility, when cells are cultured on microfluidic devices (Figure 4). By immunofluorescence and confocal analysis, we observe that primary cortical microglia cultures (Figure 5, left panel) are very different from N9 microglia cells (Figure 5, right panel) [29,30], in terms of shape and morphological adjustment to the microchannels. When residing in the culturing chamber of the microfluidic device (labeled as cc in Figures 4 and 5), between the proper microfluidic channels (labeled as mc in Figures 4 and 5) and the reservoir (round grey area in Figure 4), the majority of primary microglia (Figure 5, left panel) appears heterogeneous, with a large diameter (20–50 μm) but few and long processes (phalloidin in green; anti-P2Y12 purinergic receptor in red). Cells tend to aggregate when residing in the culturing chamber (cc) but, when present inside the microfluidic channels (mc), they extend the cytoplasm from a roundish to a stretched, elongated morphology, with a clear leading edge, similarly to what observed on line grating geometries (Figures 2D and 3B). Conversely, round-shaped N9 cells have an average diameter of about 20 μm, possess numerous very short processes and have the tendency to disorderly coalesce when residing in the culturing chamber (Figure 5, right panel, phalloidin in green, cc). When engaged inside the microfluidic channels (mc), they acquire a quadrangle shape, are strictly aligned to each other by cell contacts, as suggested by the presence of stronger phalloidin fluorescent signals at the cell-to-cell side. In the two different compartments, N9 cells however maintain quite constant their average A, AR and CR (Table 1). These parameters are instead very different in primary microglia present inside the microchannels, or in the culturing chamber. They are A: 523 ± 52 μm^2^ versus 1807 ± 545 μm^2^; AR: 20 ± 9 versus 1.36 ± 0.19; CR: 0.08 ± 0.03 versus 0.36 ± 0.12. Moreover, we also observe that both morphological appearance and average values of A, AR and CR are very different in polarized primary microglia versus not-polarized N9 cells (Table 1), especially in the microchannels, thus discouraging the use of these last for motility studies.

**Table 1:** Morphometric analysis of microglia in microfluidic devices

	**Primary microglia microchannels**	**Primary microglia microchambers**	**N9 cells microchannels**	**N9 cells microchambers**
A (μm^2^)	523 ± 52	1807 ± 545	487 ± 176	680 ± 141
AR	20 ± 9	1.36 ± 0.19	1.56 ± 0.38	1.51 ± 0.34
CR	0.08 ± 0.03	0.36 ± 0.12	0.25 ± 0.14	0.39 ± 0.2

### Primary microglia spontaneously migrate in microfluidic channels

**Figure 6:**
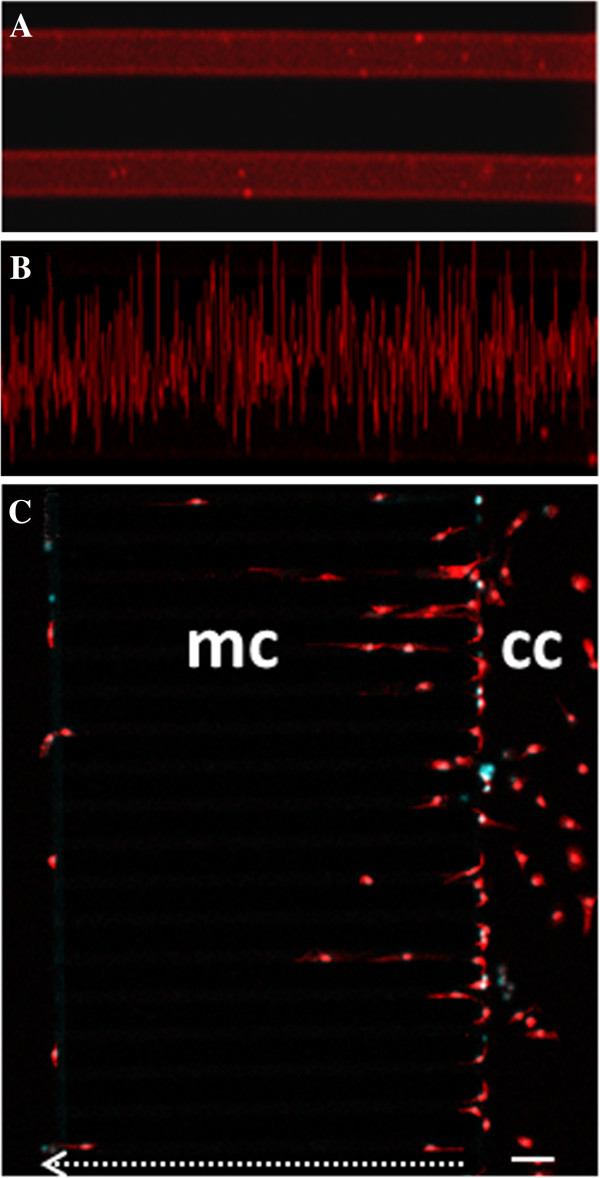
**Migration of primary microglia in microfluidic devices occurs on homogeneous fibronectin coating. A**. A homogeneous fibronectin coating is formed inside the microchannels. **B**. The profile of fluorescence intensity acquired with Zen software of Zeiss LSM 700 microscope provides values ranging from 3 to 55 fluorescence intensity arbitrary units. **C**. Microglial cells are cultured in microfluidic devices and subjected to immunofluorescence for IBA1 (red) and staining with Hoechst (blue). The total migration path is: length 500 μm (white dotted arrow), width 12 μm, height 10 μm, and the scale bar is 50 μm.

**Figure 7:**
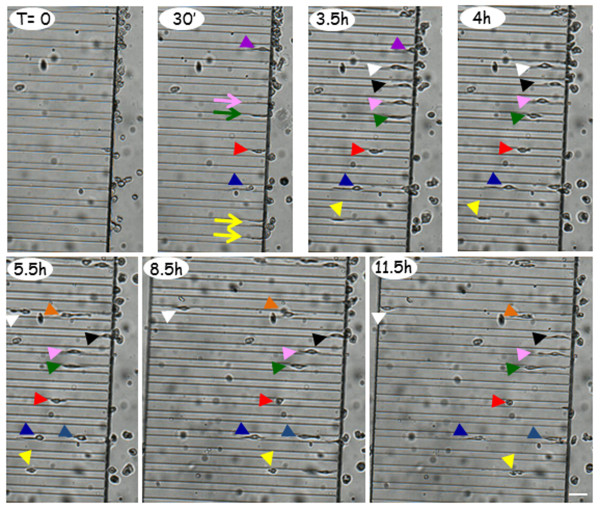
**Primary microglia spontaneously migrate in microfluidic channels.** After plating primary microglia in microfluidic devices for 30 min, time-lapse recording is performed every 30 min for 20 h. Arrows represent microglial processes and arrowheads indicate microglial cells. The different colors point to different forward- or backward-displacement of the cells inside the microchannels. The total migration path is: length 500 μm (white dotted arrow), width 12 μm, height 10 μm. The scale bar is 50 μm.

In order to prove if morphological transition affects baseline motility of primary microglia, we performed time-lapse recording on artificial microfluidic substrates with controlled characteristics (chemical composition, shape, dimensions, softness) [16]. Fibronectin-coated PDMS microfluidic microchannels with a width of 12–18 μm, a length of 500 μm (but not 3 mm), a reservoir chamber of 200 μl in volume, are found permissive to this aim (Figure 4). The presence of a fibronectin coating gradient potentially generated inside the microchannels and possibly sustaining cell migration is moreover excluded by immunofluorescence confocal analysis in the presence of anti-fibronectin (Figure 6A,B). Under these conditions, we observe that several microglial cells are scattered in the culturing chamber (cc), with many cells apparently aligned in proximity to the microchannels (Figure 6C, microglial marker IBA1 [31] in red). Moreover, we detect microglia also engaged inside the microchannels (mc), with some cells finally moving out from the microchannels, after having completed the 500 μm full length (white dotted arrow). When time-lapse is captured for 20 h every 30 minutes (Figure 7 and Additional file 1), we observe that at T = 0 min, many round-shaped microglial cells are positioned in proximity to the microchannel openings, at the boundary between microchannels and culturing chamber. At T = 30 min, several microglial processes are already surveilling the environment and entering into the microchannels (arrow), with a few cells already engaged inside the channels (arrowhead). At T = 3.5 h, several cells are committed and already moving inside the channels (arrowhead). At T = 4 h, some cells move forward (white, pink, green, red, yellow arrowhead), some stay still (black arrowhead) or move backward in the microchannels (purple arrowhead at T = 3.5 h). At T = 5.5 h, two cells (orange and light blue arrowhead) are entering in microchannels already occupied by other cells (respectively white and blue). Between T = 5.5 and T = 11.5 h, some cells change direction and move backward (pink, yellow arrowhead), some stop moving after a displacement of about 120–150 μm (green, red arrowhead) or about 250 μm (blue arrowhead). At T = 11.5 h, we also observe a cell completing the 500 μm full length and moving out from the other side (white arrowhead) (Figure 7 and Additional file 1). Moreover, we detect: cells branching and alternating their processes in and out from the same microfluidic channel, or in more than one channel; cell bodies oscillating between two adjacent channels; two cells proceeding simultaneously inside a single channel; cells inverting direction and moving out from the channels; cells overtaking each other inside the channels (Additional file 1). During the time-lapse, the cells always appear healthy and possess a roundish or elongated shape respectively in the culturing chamber and the microchannels.

### Morphometric analysis of primary microglia migrating in microfluidic channels

**Figure 8:**
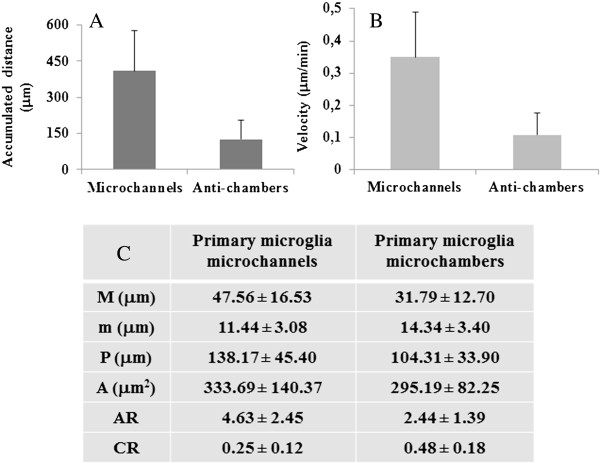
**Morphometric analysis of primary microglia migrating into microfluidic channels. A**. Mean accumulated distance of microglia somata over 20 h time-lapse recording. **B**. Mean velocity (μm/min) of microglia somata in microfluidic devices. **C**. Randomly selected cells moving in the microchannels (n = 79 cells) and culturing chamber (n = 120 cells) are manually tracked. Image stacks and time series are analyzed by Image J software. Data presented as mean ± SD. Statistical difference is obtained by Mann–Whitney test (p < 0.0003). “M” is major axis, “m” minor axis, “P” cell perimeter, “A” cell area, “AR” aspect ratio, “CR” circularity ratio.

Quantitative analysis of individual somata performed during the time-lapse confirms the highly dynamic nature of microglia. By analyzing image stacks and time series with Image J software, we observe that: a) only 2 ~ 3 cells are found simultaneously inside a single microchannel; b) the percentage of total cells engaged in the microchannels is about 50% of those residing in the culturing chamber; c) the maximal distance covered by a single cell in the microchannel is 500 μm in 11.5 h; d) the average accumulated distance of microglia somata over 20 h recording is 409.2 ± 169.6 μm in the microchannels, with respect to 125.6 ± 79.5 μm in the culturing chamber (Figure 8A); e) the highest velocity is 0.66 μm/min; f) the mean velocity of microglia somata inside the microchannels is 0.350 ± 0.145 μm/min, as compared to 0.107 ± 0.068 μm/min in the culturing chamber (Figure 8B). Moreover, also microglia processes (defined as cytoplasm protrusion with a length equal to at least one cell body diameter) are found extremely motile, undergoing extensions and retractions when moving inside the microchannels. The maximal length change of individual processes inside the microchannels is 70–80 μm. To quantify the morphological changes, we analyzed cells present in the different compartments of the microfluidic device (Figure 8C). Inside the microchannels (n = 79 cells), we observe a 50% decrease in the average CR (0.25 ± 0.12), with respect to cells (n = 120) in the culturing chamber (CR = 0.48 ± 0.18), while the average AR increases about twofold.

## Discussion

Microglia consist of at least two subpopulations that coexist in the adult CNS and derive from different sources: one that originates from bone marrow-derived cells and migrates to the CNS during embryonic development for colonization of the nervous system parenchyma, the second that develops from myeloid progenitor cells and enters the brain after birth [32], becoming fundamental for microglial maintenance of the CNS homeostasis [6,33]. Since adult microglia can play a twofold role, either amplifying the effects of inflammation and mediating cell degeneration, or protecting the nervous system from pathological insults [6], efficient chemotaxis can acquire either neuroprotective or inflammatory and detrimental roles. Whereas we are currently capable of distinguishing amoeboid-phagocytic from branched-surveilling microglia [3,5], we are unaware of how these morphological shapes can denote a neuroprotective or neurotoxic role. In other words, it is still an open matter how the vast morphological heterogeneity existing within the mixed microglia population might relate to functional diversification.

Cell behavior strictly depends on the stiffness and shape of the microenvironment, and the response to surface topography is a very critical determinant of cell morphogenesis. Microglia respond to micro- nano-structured pits, protrusions and grooves with altered morphology, adhesion, and directional growth [19]. In our work, we establish that primary microglia retain *in vitro* the intrinsic competence of modifying their structure in response to contact guidance cues. When grown on pillar-shaped substrates, microglia acquire a center-stage multi-polar morphology and develop abundant button-like pseudopodia emerging from the cell body with a nearly radial orientation. This situation might very well depict the *in vivo* condition of microglia not committed to migrate, but exploring the environment with short forward, backward and sideways steps, with the final purpose to orient their migration. When placed on a line-grating substrate in the presence of parallel and symmetrical grooves, microglia elicit a longitudinally flattened appearance with elongated bipolar shape. This *in vitro* condition might favor a polarized extension of filopodia at the leading edge of the cell, in preparation of a forward translocation of the cell body, with retraction of the rear of the cell [26]. The adjustment of microglia to a line-grating geometry is thus highly suggestive of commitment to follow a straight path *in vivo*. Thus, surface topography can induce a synchronous and homogeneous metamorphosis of microglia *in vitro*, distinguishing the center-stage multipolar (on micropatterned pillars) from the elongated bipolar (on line-grating micropatterns) cells. With the use of specific markers and cytoskeleton-perturbing drugs, future work will aim to establish if these subpopulations can be correlated to: a) microglia generated from bone marrow rather than from myeloid progenitor cells; b) microglia colonizing the CNS during development, rather than maintaining CNS homeostasis in adulthood; c) more importantly, “beneficial” rather than “detrimental” microglia. Moreover, future analysis of parameters such as cell adhesion, distribution of actin cytoskeleton and microtubules, phagocytosis, antigen presenting power, beneficial or detrimental chemokine/cytokine expression and release, will allow to determine the functional identity of multipolar versus bipolar microglia.

Moreover, by the use of bio-mimetic 3D microfluidic substrates, our work demonstrates that primary microglia appear intrinsically different from immortalized N9 cells. During cell polarization inside the microchannels, primary microglia tend to align longitudinally and individually, whereas immortalized N9 microglia assume a more compact morphology with the propensity to establish cell contacts. This might be reminiscent of the immortalization program that likely confers to N9 cells a compact morphology optimizing cell division and contact formation, rather than the aptitude of primary cells to explore the environment with a direction-oriented polarization. In both cases, specific receptors and structural molecules are found enriched respectively at the cell-to-cell surface (N9 cells) or leading edges (primary microglia), as evinced by increased phalloidin and P2Y12 receptor signals at these sites.

Cell adhesion and morphology are dynamically mutable during cell migration [22]. The last issue that we addressed in our work is thus the free motility of microglia in culture. While numerous publications cite motility and short migration of microglia *in vivo*, only few works describe microglia moving for long distances. This is the case for example of the work by Carbonell and coauthors [34], demonstrating by intracerebroventricular injection of rhodamine and time-lapse confocal microscopy that subventricular microglia at the interface of the cerebrospinal fluid and brain parenchyma, exhibit the “in situ” ability to migrate for several hundred microns into the parenchyma, towards a deafferentation injury of the hippocampus. Conversely, Nimmerjahn and colleagues indicate only static movement of microglia, branch motility, but not cell bodies migration [2]. Using transcranial two photon microscopy, Davalos and coauthors [8] confirm that only microglial processes are highly dynamic in intact brain, reaching up to several micrometers in length, or retracting until they completely disappear. This baseline dynamism of microglial processes is thus in sharp contrast to the stability of the microglial cell bodies and surrounding neuronal processes [4].

*In vitro* studies on this subject have instead shown only transwell device infiltration directed by a chemical gradient. Lee and Chung [35] describe that ADP stimulates chemotaxis of immortalized BV2 microglia from the upper to the lower side of the transwell membrane. Karlstetter and co-authors [36] present evidence that curcumin inhibits basal and LPS-induced relocation of BV2 microglia from the upper to the lower transwell membrane surface [13]. However this passage across a physical barrier limits the possibility of investigating long distance migrations and, moreover, provides only indirect evaluation of kinetic parameters. Honda and collaborators [37] using the Dunn chemotaxis chambers report that primary rat microglia have weak motility in the absence of ligands, but perform a mean displacement of about 50 μm in the presence of ATP or ADP. Nasu-Tada et al. [38] confirm that primary microglia are almost static in the absence of stimulants, but show chemotactic responsiveness to ADP with a maximal displacement of about 140 μm. Finally, Haynes and coauthors [39] prove that the leading edge of mouse primary microglia moves for a maximal displacement of 50 and 120 μm after 30 minutes, in a gradient of ATP or ADP, respectively. However, the average distance spontaneously migrated in the absence of any provided stimulus is only 0.8 μm.

All considered, spontaneous long distance migration of microglia is described *in vivo* with conflicting results, but never *in vitro* until now. Here, we demonstrate that biocompatible polimeric channels constitute a more suited environment than transwell devices or Dunn chemotaxis chambers for permitting and measuring spontaneous long distance migration *in vitro*. Indeed, the 500 μm traveled by primary microglia inside the microfluidic channels in the absence of stimuli are much above the known average distance so far reported and this achievement might be perhaps improved.

In summary, by the use of interdisciplinary techniques, our data indicate that microfluidic technologies and microstructured patterns with control over the presentation of adhesion sites, are able to sort out different morphological structures within a mixed microglia population and allow free motility *in vitro*. By reproducing a specialized niche for the cells, these technologies can help to understand the role of structural determinants in priming morphogenesis and free motility of microglia and may be exploited for translational research on functional tissue engineering and implantable device design.

## Conclusions

With our work we have shown that a strict control over biomaterial surface topography by soft lithography techniques can highly impact on microglia morphology and greatly improve spontaneous motility *in vitro*. The use of microstructured 3D devices to manipulate microglia is thus of interest to scientists working on the mechanisms of cell substrate/matrix interactions, morphogenesis and migration, and particularly on microglia responses and functions also during neurodevelopmental and neuroinflammatory disorders. The advantage of using primary microglia in microfluidics and micropatterning opens the road to the use of these devices for testing drug candidates for CNS neuroinflammatory diseases.

## Methods

### Reagents

All reagents for cell culture are obtained from Sigma-Aldrich, unless otherwise stated. The culture media DMEM and DMEM-F12 are acquired from Invitrogen. Fetal bovine serum (FBS) is obtained from Gibco.

### Fabrication and preparation of microstructured PDMS substrates

Microtopographic silicon masters are realized to replica-mold substrates on PDMS (Sylgard 184, Dow Corning) with line-grating and pillar-shaped geometries. The micropatterns dimensions in both cases are width: 1500 nm, pitch: 3 μm, height: 550 nm. The fabrication process is started with the patterning of PMMA by 100 kV e-beam lithography on a Si substrate. A 20 nm Cr film is evaporated by electron gun followed by lift-off process in acetone at 50°C and sonication. Samples are then etched up to 550 nm by Reactive Ion Etching using CHF_3_, O_2_, SF_6_ and Ar gas mixtures. After Cr wet etching, Si substrates are thermally oxidized at 950°C for 2 h in O_2_ atmosphere to reduce surface roughness and then immersed in the hydrofluoric acid buffer to remove the thin oxide layer. After cleaning in Piranha solution (H_2_SO_4_/H_2_O_2_, 3:1), microstructured Si wafers are silanized with 10% trimethylchlorosilane in toluene in N_2_ environment to generate a low-energy surface and facilitate the subsequent mold-PDMS separation. The micropatterns are reproduced on PDMS by standard soft lithographic techniques. A 10:1 (v/v) mixture of monomer and curing agent is prepared and diluted in a 5% (v/v) solution in n-heptane, in order to lower the viscosity. The mixture spin-coated onto the Si master mold is left undisturbed for 2–3 h to allow the solution to penetrate into the voids of the master and for solvent evaporation. Then, it is cured for 30 min at 60°C. Onto this thin PDMS layer, a liquid prepolymer of Sylgard 184 PDMS 10:1 (v/v) is poured and degassed. Then, it is reticulated on a hotplate for 1 h at 60°C and peeled off from the mold. Micropatterned PDMS samples are plasma oxidized and kept in water before cell culture, in order to produce and maintain a hydrophilic surface.

### Fabrication and design of microfluidic devices for real-time cell analysis

Standard soft lithography procedures are performed to fabricate all microdevices in PDMS, a biocompatible thermo-curable elastomer [40,41]. The microfluidic device features two cell culture compartments (1 mm wide, 7 mm in length and 100 μm high) connected via a set of micron-size channels each with dimensions: width = 12 or 18 μm (depending on the experiment), length = 500 μm, height = 10 μm (Figure 4). The circular wells (8 mm in diameter) serve as loading inlets and cell medium reservoirs for nutrient and gas exchange. The design configuration was adapted from Hosmane and collaborators [42], optimizing microchannel dimensions to the microglia physical scale. The master molds are realized by two-layer microfabrication process using the negative photoresist SU-8 (MicroChem Corp, Newton, MA). Briefly, patterns for standard photolithography are designed with CAD software and transferred on two chrome masks by electron-beam lithography. Silicon wafers are spin coated with a layer of SU-8 3005 at a rate of 1000 rpm (resist thickness 10 μm), pre-baked at 95°C for 3 min, exposed to a i-line (365 nm) UV light source for 16 s through a photo mask (with the microchannel pattern and alignment marks) and post-baked (1 min at 65°C, 3 min at 95°C). Next, the second photolithography step on Su-8 3050 (100 μm thick) transfers the chamber areas and reservoirs aligned to the first pattern (pre-bake: 45 min at 95°C, exposure time: 18 seconds, post-bake: 1 min at 65°C, 5 min at 95°C). PDMS (Sylgard 184, Dow Corning) chips are obtained by replica molding, casting the prepolimer base and cross-linker at the volume ratio of 10:1, over the patterned master template. After degassing for 30 minutes in a vacuum chamber the PDMS is allowed to polymerize at 120°C on a hotplate for 1 h. Once cross-linked, it is carefully released from the mold and then fluidic access ports are created using a suite of 8 mm dermal biopsy punch tools (Kai Medical). To form an irreversible bonding, the surfaces of PDMS replica and microscope glass slides are O_2_ plasma-activated (Oxford Plasma Lab 80 plus, RF Power: 20 W, Flux: 60 sccm, Pressure: 700 mtorr, Time: 30 s) and put in contact immediately after exposure. Assembled devices are post-baked at 70°C for 2 h to complete and enhance adhesion strength. Before plasma treatment bonding, microscope glass slide (52 mm × 76 mm, Menzel-Glaser) are cleaned in Piranha solution (H2SO4/H2O2 3:1), rinsed in DI water and dried with N2 gun to remove debris and other surface contaminants.

### Coating of micropatterned structures with fibronectin

After UV sterilization for 20 min, the micropatterned devices are kept immersed in a solution of 10 μg/ml fibronectin (Sigma-Aldrich) for 1 h at room temperature, followed by three washes with sterile water.

### Coating of microfluidic devices with fibronectin

After UV sterilization for 20 min, the coating of microfluidic devices is performed according to Park and coauthors [43]. Each reservoir is loaded with 100 μl of 10 μg/ml fibronectin (Sigma-Aldrich) and the entire device is allowed to be filled. The coating solution is kept for 1 h at room temperature and three washes are performed after removal of the fibronectin solution. Homogeneity of fibronectin coating is verified by indirect immunofluorescence, in the presence of anti-fibronectin (Calbiochem) used at a dilution of 1:100 in phosphate buffer saline (PBS, 24 h at 4°C), followed by rabbit anti-goat rhodamine conjugated antiserum (3 h at 24°C). Confocal analysis is performed (as described below), and the profile of fluorescence intensity is obtained with the Zen software of Zeiss LSM 700 microscope.

### Primary cortical microglia

All animal procedures have been performed according to the European Guidelines for the use of animals in research (86/609/CEE) and the requirements of Italian laws (D.L. 116/92). The ethical procedure has been approved by the Animal Welfare Office, Department of Public Health and Veterinary, Nutrition and Food Safety, General Management of Animal Care and Veterinary Drugs of the Italian Ministry of Health. All efforts were made to minimize animal suffering and to use the number of animals only necessary to produce reliable results. Primary microglial cultures are prepared from 1 to 2 day-old rat and mouse, as previously described by Chen and collaborators [44]. In brief, after removing the meninges, cortices are minced and digested with 0.01% trypsin and 10 μg/ml DNase I. After dissociation and passage through 70-μm nylon cell strainer (BD Biosciences Europe), cells are resuspended in DMEM medium supplemented with 20% heat-inactivated FBS, 4 mM glutamine, 1 mM sodium pyruvate, 50 U/ml penicillin, 50 μg/ml streptomycin, 100 μg/ml gentamicin and plated in T75 poly-D-lysine-coated flasks, at about 10 million cells/flask. The cultures are kept at 37°C in a 5% CO2 and 95% air atmosphere. Every 2–3 days, the medium is entirely changed for the next 12-days. At about 14 days after plating, mixed glial cultures are shaken at 200 rpm at 37°C for 1 hour. The microglial cells are collected from each flask and plated at different density on microfluidic devices or micropatterned supports coated with 10 μg/ml fibronectin. A population 99% pure of microglial cells is obtained as verified by immunofluorescence with GFAP (for astrocytes), NeuN (for neurons), NG2 (for oligodendrocytes) and CD11b clone OX42 (for microglia).

### Microglial N9 cell line

The murine N9 microglia cell line is grown in DMEM-F12 medium supplemented with 10% heat-inactivated FBS, 4 mM glutamine, 50 U/ml penicillin, 50 μg/ml streptomycin and 100 μg/ml gentamicin. The N9 microglia is kept at 37°C in a 5% CO2 and 95% air atmosphere.

### Cell loading

Loading of the cells in the microfluidic devices is performed mainly according to Park and coauthors [43]. Briefly, the cell device is maintained filled with 200 μl of culture medium for 1 h in a humidified incubator, before plating the cells. The medium is then removed from the reservoirs and 1×10^4^ cells are immediately seeded. The device is kept in the incubator for 15–20 min to allow cell adhesion and, after replacement of fresh media in the reservoirs, the device is then maintained at 37°C in a 5% CO2 and 95% air atmosphere, until used. Plating density is set in the range of 125 cells/mm^2^.

### Immunofluorescence using micropatterned structures

Microglia are washed three times with PBS, fixed with 4% paraformaldehyde for 20 min, washed, permeabilized with 0.05-0.1% Triton X-100 for 10 min, rinsed, blocked for 30 min in 1% PBS/BSA (bovine serum albumin), and stained with 5 μg/ml Cy2-phalloidin (Sigma-Aldrich) alone, or in combination with the primary antiserum against P2Y12 receptor (Anaspec) used at 1:100 diluition, in 1% PBS/BSA, for about 3 h at 37°C, or stained with anti-paxillin (BD Biosciences) used at 1:1000. The secondary antibodies used for double labeling are Cy3-conjugated donkey anti-rabbit IgG (1:100, Jackson Immunoresearch) or Cy2-conjugated donkey anti-mouse IgG (1:100, Jackson Immunoresearch). The cells are then extensively washed and stained with the nucleic acid blue dye, Hoechst 33342 (1:1000). After rinsing, the cells are covered with gel/mount™ anti-fading medium (Biomeda Corporation) and a coverslip. Incorporated fluorescence is analyzed by means of a fluorescence microscope (Olympus BX51). Brightness and contrast of digital images are adjusted using Microsoft Office PowerPoint 2007.

### Immunofluorescence using microfluidic devices

Microglia is washed three times by loading each reservoir with 200 μl PBS. Cells are then fixed with 4% paraformaldehyde for 20 min, washed, permeabilized with 0.05-0.1% Triton X-100 for 15 min, rinsed, blocked for 30 min in 1% PBS/BSA, and stained with 5 μg/ml Cy2-phalloidin (Sigma-Aldrich), alone or in combination with the primary antiserum against P2Y12 receptor (Anaspec) used at 1:100 dilution, or with IBA1 (Wako Chemicals GmbH) at 1:200, in 1% PBS/BSA, for 24 h at 4°C. The secondary antibody used for double immunofluorescence is Cy3-conjugated donkey anti-rabbit IgG (1:100, Jackson Immunoresearch). Finally, the cells labeled with IBA1 are also extensively washed and allowed to incorporate the nucleic acid blue dye, Hoechst 33342 (1:1000).

### Confocal microscopy

After rinsing of the cells, double or triple incorporated immunofluorescence is analyzed by means of a confocal laser scanning microscope (LSM 700, Zeiss) equipped with four laser lines: 405 nm, 488 nm, 561 nm and 639 nm. The brightness and contrast of the digital images are adjusted using Microsoft Office PowerPoint 2007.

### Time-lapse microscopy

Primary microglia are recorded with objective 4× at regular time-lapse (30 min) over a 20 h period, with a bright-field microscope equipped by a time-lapse system (JuLI, Digital Bio). The images are captured with CMOS 1.3 M pixels camera and exported as TIFF files.

### Image processing

Fluorescence microscopy images are imported into Matlab, converted into binary 8-bit images and processed by a code developed according to Kozlowsky and Weimer [45]. The images are then subjected to quantitative analysis of morphometric parameters. Tracking analysis of time-lapse microphotographs is performed by Image J manual tracking plugin. The generated tracking data are used to calculate microglia motility parameters. Audio Video Interleave (AVI) files are generated by using the entire time-lapse image sequence with Image J software (Additional file 1).

### Statistical analysis

The Mann–Whitney test is used for non-parametric analysis of differences between groups. P <0.05 is considered statistically significant.

## Abbreviations

A: Cell area; AR: Axis ratio; BSA: Bovine serum albumin; CNS: Central nervous system; CR: Circularity ratio; 3D: Three-dimensional; FBS: Fetal bovine serum; M: Major axis; m: minor axis; P: Cell perimeter length; PBS: Phosphate buffer saline; PDMS: Poly(dimethylsiloxane).

## Competing interests

The authors declare that they have no competing interests.

## Authors’ contributions

CV and LB conceived and designed the study. LB and AG designed microstructured PDMS substrates and microfluidic devices for cell culturing. AD provided fabrication and preparation of microstructured PDMS substrates and microfluidic devices. SA performed primary microglia purification, culturing on microstructured substrates and microfluidic devices, and real time cell analysis. SA and CM completed confocal fluorescence analysis. LB, AD and AG gathered and analyzed data. CV and SA wrote the article, which was revised by LB and AG. All authors read and approved the final version of manuscript.

## Supplementary Material

Additional file 1 **The video shows baseline motility of primary microglia for long distances on artificial bio-mimetic microfluidic substrates with controlled physical/chemical characteristics.** We performed regular 30 min time-lapse recording (JuLI, Digital Bio) over a 20 h period. Primary microglia spontaneously travel through fibronectin-coated PDMS microfluidic channels (width 12 μm and length 500 μm) for a distance of up to 500 μm in approximately 12 h, with an average speed of 0.66 μm/min. Click here for file
